# Supplementary material for: Dendropanoxide, a Triterpenoid from Dendropanax morbifera, Ameliorates Hepatic Fibrosis by Inhibiting Activation of Hepatic Stellate Cells through Autophagy Inhibition
Source: Nutrients. 2021 Dec 27;14(1):98. doi: 10.3390/nu14010098 (PMC8796030; doi:10.3390/nu14010098)
Supplement: Supplementary file 1 [file nutrients-14-00098-s001.zip › nutrients-1494448-supplementary.pdf]

Supplementary Materials

A

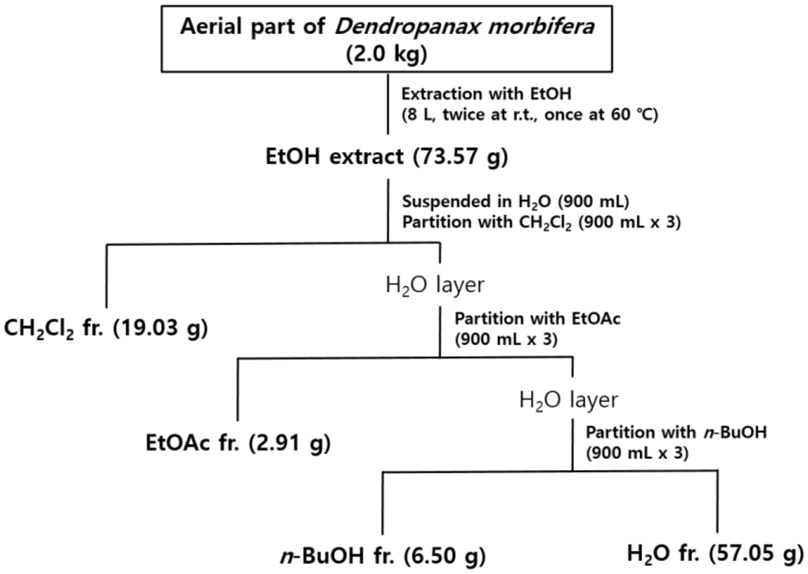

B

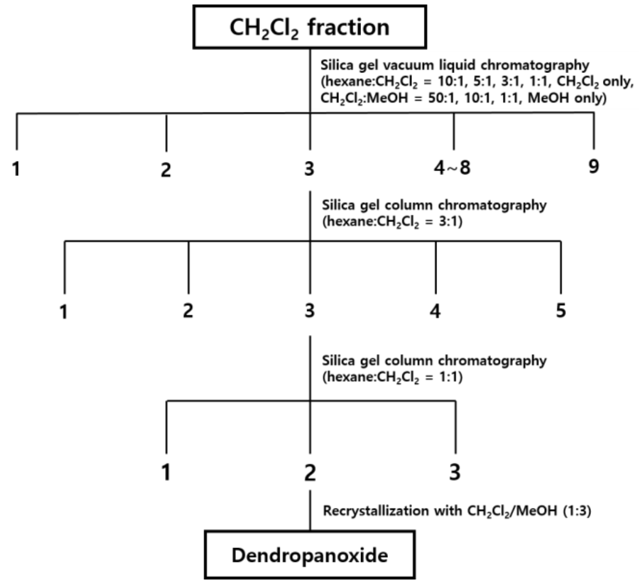

Figure S1. Extraction and isolation scheme for dendropanoxide from the aerial parts of *Dendropanax morbifera*. (A) Extraction and partition scheme for the aerial parts of *D. morbifera*. (B) Scheme for the isolation of dendropanoxide from the dichloromethane fraction.

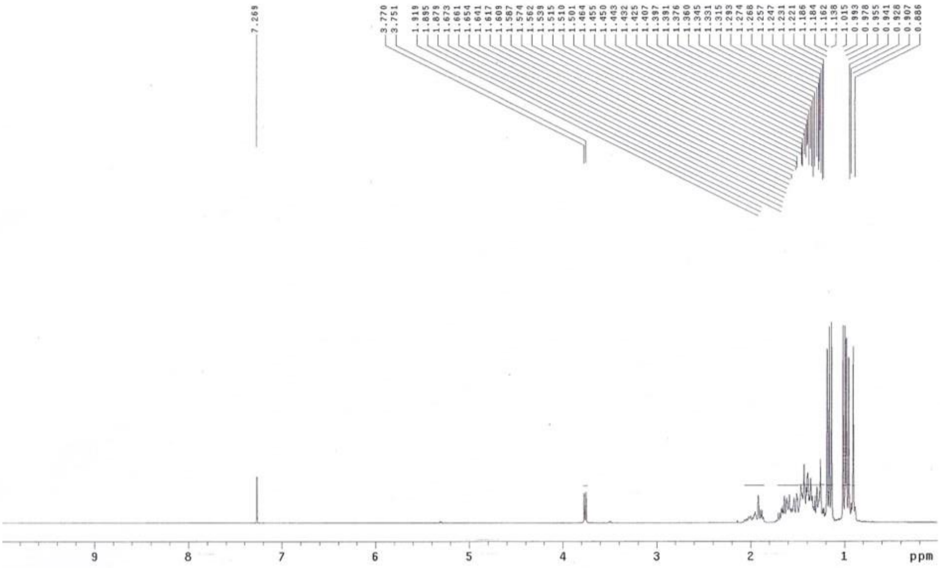

Figure S2. ^{1}H NMR spectrum of dendropanoxide ($CDCl_3$, 500 MHz).

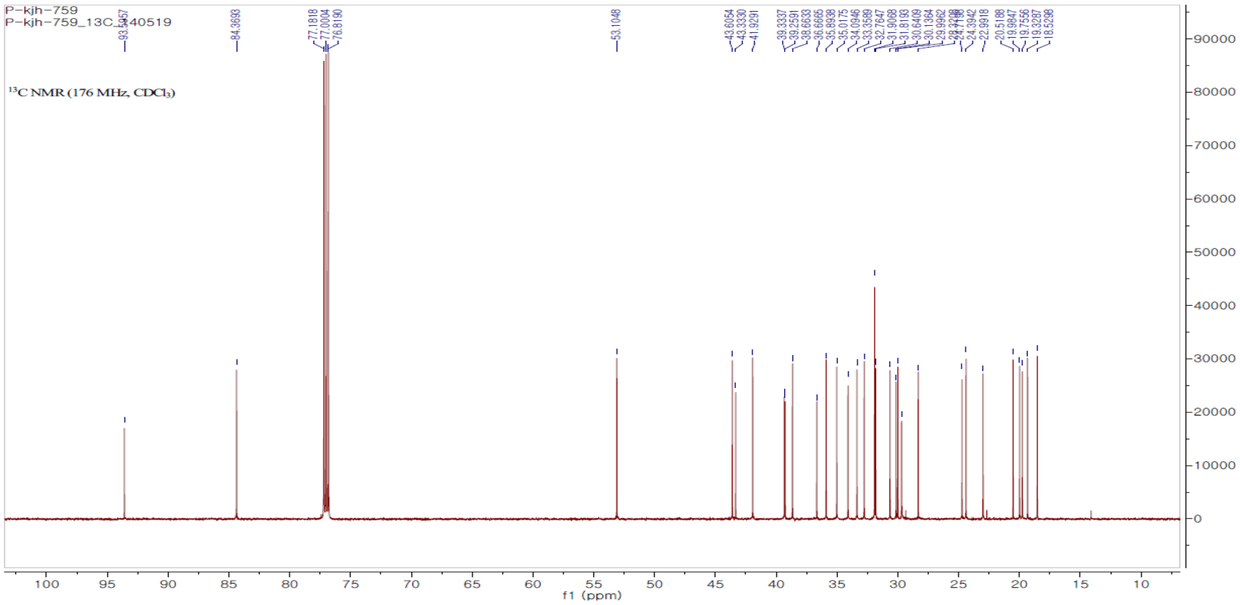

Figure S3. ^{13}C NMR spectrum of dendropanoxide ($CDCl_3$, 125 MHz).

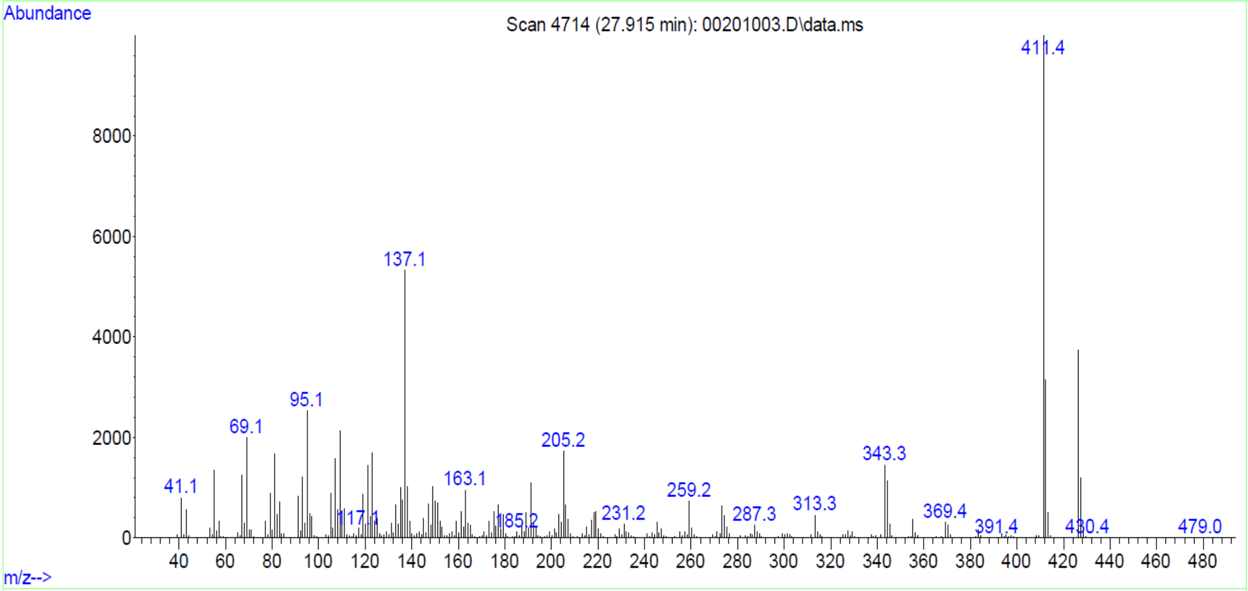

Figure S4. GC/EIMS spectrum of dendropanoxide.
